# Chromosomal Heterogeneity and Enrichment of Mitotic Regulatory Programs Reveal a Therapeutic Vulnerability in Cytarabine-Resistant FLT3-ITD AML

**DOI:** 10.3390/cimb48090966

**Published:** 2026-09-21

**Authors:** Jui-Hung Yen, Zi-An Chen, Yu-Xuan Lin, Hui-Yu Jiang, Liang-In Lin, Chi-Cheng Li, Pei-Yi Chen

**Affiliations:** 1 Department of Molecular Biology and Human Genetics, Tzu Chi University, Hualien 970374, Taiwan; imyenjh@gms.tcu.edu.tw (J.-H.Y.); 109727106@gms.tcu.edu.tw (Z.-A.C.); 110712122@gms.tcu.edu.tw (Y.-X.L.); 2 Laboratory of Medical Genetics, Genetic Counseling Center, Hualien Tzu Chi Hospital, Buddhist Tzu Chi Medical Foundation, Hualien 970374, Taiwan; huiyuj26@gmail.com; 3 Department of Clinical Laboratory Sciences and Medical Biotechnology, College of Medicine, National Taiwan University, Taipei 10048, Taiwan; lilin@ntu.edu.tw; 4 Division of Hematology-Oncology, Department of Internal Medicine, Hualien Tzu Chi Hospital, Buddhist Tzu Chi Medical Foundation, Hualien 970374, Taiwan; kevinlcc1234@gmail.com

**Keywords:** cytarabine, AML, FLT3-ITD, chromosomal heterogeneity, mitotic spindle

## Abstract

Cytarabine is a cornerstone of acute myeloid leukemia (AML) therapy; however, acquired resistance remains a major clinical challenge. FMS-like tyrosine kinase 3 internal tandem duplication (FLT3-ITD), a common genetic alteration in AML, is associated with high relapse rates and poor outcomes. Here, we investigated the cellular and molecular mechanisms of acquired cytarabine resistance in FLT3-ITD AML. Cytarabine-resistant MV4-11-CR and MOLM-13-CR cells were generated from parental MV4-11 and MOLM-13 cells, respectively, by stepwise drug selection. Both models exhibited markedly elevated cytarabine IC_50_ values and enhanced proliferation. FLT3 expression and activation increased in MV4-11-CR cells but decreased in MOLM-13-CR cells. Midostaurin pretreatment failed to restore cytarabine sensitivity, indicating that altered FLT3 signaling is not a shared mechanism of resistance. Cytogenetic analyses and interphase FISH revealed greater numerical and structural chromosomal heterogeneity in the resistant cells than in their parental counterparts, including the presence of polyploid and near-tetraploid subpopulations. RNA sequencing identified common transcriptional alterations, with *DCK* among the most significantly downregulated genes. Reduced *DCK* expression was confirmed at the mRNA and protein levels. *DCK* knockdown in parental MV4-11 cells attenuated cytarabine-induced cytotoxicity, whereas its re-expression in MV4-11-CR cells partially restored cytarabine sensitivity, supporting the functional contribution of *DCK* downregulation to acquired resistance. Gene set enrichment analysis demonstrated shared enrichment of G2/M checkpoint and mitotic spindle pathways. Notably, combined TTK/Mps1 and FLT3 inhibition with luvixasertib and midostaurin synergistically suppressed cell viability in both resistant cell lines. These findings identify divergent FLT3 regulation, *DCK* downregulation, increased chromosomal heterogeneity, and enrichment of mitotic regulatory programs as key features associated with acquired cytarabine resistance. They also support the combined inhibition of TTK/Mps1 and FLT3 as a potential therapeutic strategy for cytarabine-resistant FLT3-ITD AML.

## 1. Introduction

Acute myeloid leukemia (AML) is an aggressive hematologic malignancy characterized by the clonal expansion of immature myeloid progenitor cells and impaired hematopoietic differentiation. Cytogenetic and molecular abnormalities are central to AML classification, risk stratification, and therapeutic decision-making [1,2]. Although targeted therapies have improved outcomes in selected molecular subgroups, conventional chemotherapy remains a major component of AML treatment, and disease relapse driven by acquired drug resistance continues to be a major cause of treatment failure [3,4].

Cytarabine (Ara-C) is a key component of AML induction and consolidation therapy. Following cellular uptake, cytarabine undergoes sequential phosphorylation to form its active metabolite, cytarabine triphosphate (Ara-CTP), which is incorporated into replicating DNA and inhibits DNA synthesis [5,6]. Deoxycytidine kinase (*DCK*) catalyzes the initial and rate-limiting phosphorylation of cytarabine, thereby playing a critical role in determining intracellular Ara-CTP accumulation and cytotoxic response [7]. Reduced drug uptake, impaired cytarabine activation, enhanced drug inactivation, and altered DNA damage responses have all been implicated in cytarabine resistance [8]. However, these established mechanisms may not fully account for the broader cellular adaptations that emerge during prolonged cytarabine exposure.

FMS-like tyrosine kinase 3 (FLT3) is frequently altered in AML, with internal tandem duplication (FLT3-ITD) mutations among the most common recurrent oncogenic lesions [9,10,11,12]. Constitutive FLT3-ITD signaling promotes leukemic proliferation and survival and is associated with increased relapse risk and adverse clinical outcomes [13,14]. FLT3-ITD has also been linked to replication stress, DNA damage, and genomic instability, suggesting that sustained oncogenic signaling may facilitate genetic diversification during treatment [15,16]. Nevertheless, FLT3-targeted inhibition does not uniformly eliminate chemotherapy-resistant cells, indicating that resistant FLT3-ITD AML may acquire additional survival mechanisms beyond persistent FLT3 activation.

Chromosomal instability (CIN) generates numerical and structural chromosome alterations, thereby increasing tumor heterogeneity and facilitating clonal adaptation under therapeutic pressure [17]. Polyploidization and mitotic dysregulation may further support the survival and evolution of drug-resistant cancer cells. Conversely, cells with increased chromosomal heterogeneity may become increasingly dependent on mitotic regulatory mechanisms, particularly the spindle assembly checkpoint (SAC), to preserve sufficient chromosome segregation fidelity for continued proliferation. TTK/Mps1 is a central SAC kinase and has emerged as a potential therapeutic target in genomically unstable cancers [18,19].

Although FLT3 inhibitors have improved outcomes in FLT3-mutated AML, relapse, acquired resistance, and treatment-related toxicities continue to limit durable responses. Given the potential dependence of chromosomally heterogeneous cells on spindle assembly checkpoint activity, combined TTK/Mps1 and FLT3 inhibition may provide a mechanistically distinct strategy for targeting cytarabine-resistant FLT3-ITD AML. However, this combination has not been systematically investigated in the context of acquired cytarabine resistance. In this study, we investigated whether cytarabine-resistant FLT3-ITD AML cells acquire enhanced proliferative capacity and shared chromosomal and transcriptomic alterations despite heterogeneous FLT3 regulation. We further examined potential contributors to cytarabine resistance and evaluated combined TTK/Mps1 and FLT3 inhibition as a therapeutic strategy for targeting the resistant phenotype.

## 2. Materials and Methods

### 2.1. Chemicals

Cytarabine, dimethyl sulfoxide (DMSO), MTT, ethidium bromide, RPMI 1640 medium, non-essential amino acids, and other chemicals, unless otherwise specified, were purchased from Sigma-Aldrich (St. Louis, MO, USA). Midostaurin and luvixasertib (CFI-402257) were purchased from MedChemExpress (Monmouth Junction, NJ, USA).

### 2.2. Cell Culture

The parental human FLT3-ITD-positive MV4-11 and MOLM-13 cell lines were provided by Prof. Liang-In Lin (National Taiwan University). These cell lines were originally obtained from the German Collection of Microorganisms and Cell Cultures (DSMZ, Braunschweig, Germany). Cells were cultured in RPMI 1640 medium supplemented with 10% fetal bovine serum (FBS; Thermo Fisher Scientific, Rockford, IL, USA) and 1% non-essential amino acids at 37 °C in a humidified atmosphere containing 5% CO_2_ [20,21]. The cytarabine-resistant MV4-11-CR cells were established in the laboratory of Prof. Liang-In Lin and subsequently provided to our laboratory. Their establishment and authentication by short tandem repeat (STR) profiling have been described previously [22]. MOLM-13-CR cells were established in our laboratory through stepwise exposure of parental MOLM-13 cells to increasing concentrations of cytarabine, beginning at approximately the IC_50_ of the parental cells. The cells were maintained at each concentration for at least 2 weeks, and the cytarabine concentration was increased approximately twofold once sustained cell proliferation was observed. The overall selection process lasted approximately 3-6 months. Resistance was confirmed using the MTT assay. After establishment, the resistant cells were cryopreserved and subsequently maintained in cytarabine-free medium. The resistant phenotype remained reproducible after thawing and retesting.

### 2.3. Analysis of Cell Viability and Proliferation

Cell viability was assessed using the MTT assay as previously described [23]. Briefly, cells were treated with vehicle or the indicated concentrations of cytarabine, midostaurin, luvixasertib, or their combinations for the specified durations. Unless otherwise indicated, the final DMSO concentration did not exceed 0.1%. Following treatment, cells were incubated with MTT (1 mg/mL) for 3 h at 37 °C. The resulting formazan-containing cell pellets were collected and dissolved in 0.5 mL DMSO. Absorbance was measured at 550 nm using a microplate reader, and cell viability was expressed relative to the corresponding vehicle-treated control. For proliferation assays, cells were seeded at 5 × 10^4^ cells per well in 0.5 mL of culture medium in 24-well plates. Viable cells were counted after 24, 48, 72, and 96 h using the trypan blue exclusion method [24].

### 2.4. Reverse-Transcription Quantitative Polymerase Chain Reaction (RT-qPCR)

Total cellular RNA was extracted using the Blood/Cultured Cell Total RNA Purification Mini Kit (Favorgen Biotech, PingTung, Taiwan) according to the manufacturer’s instructions. Complementary DNA (cDNA) was synthesized using the High-Capacity cDNA Reverse Transcription Kit (Thermo Fisher Scientific). The RT-qPCR reaction mixture consisted of cDNA, specific primers (Table S1), and Maxima™ SYBR Green/ROX qPCR Master Mix (Thermo Fisher Scientific). Reactions were conducted using a Roche LightCycler 480 Real-Time PCR System (Roche Diagnostics, Rotkreuz, Switzerland). Gene expression was calculated using the 2^−ΔΔCt^ method and normalized to human β-actin mRNA expression. Experiments were performed using three independent biological replicates, with each RT-qPCR reaction conducted in technical triplicate.

### 2.5. Western Blot Analysis

Total cellular proteins were extracted using RIPA buffer supplemented with Halt Protease Inhibitor Cocktail (Thermo Fisher Scientific). Protein concentrations were determined using the Bradford assay (Bio-Rad Laboratories, Hercules, CA, USA). Equal amounts of protein were separated by 10% SDS-PAGE and transferred onto polyvinylidene difluoride membranes (Cytiva, Marlborough, MA, USA). Membranes were incubated with primary antibodies against FLT3 (cat. #3462), phosphorylated FLT3 (cat. #3464), β-actin (cat. #8457S; Cell Signaling Technology, Danvers, MA, USA), and *DCK* (cat. ab96599; Abcam, Cambridge, UK), followed by horseradish peroxidase-conjugated secondary antibodies (cat. #GTX213110-01; GeneTex, Irvine, CA, USA). Protein bands were detected using ECL Select Western Blotting Detection Reagent (Cytiva) and visualized using Amersham Hyperfilm ECL (Cytiva) or an iBright FL1500 Imaging System (Thermo Fisher Scientific). Band intensities were quantified using ImageJ software (version 1.54d; National Institutes of Health, Bethesda, MD, USA), normalized to β-actin, and expressed relative to the corresponding control. Data were obtained from at least three independent experiments.

### 2.6. Chromosomal Karyotype and Fluorescence in Situ Hybridization (FISH)

Chromosome analysis was performed as previously described [25]. Metaphase chromosomes were prepared and analyzed using conventional G-banding, and karyotypes were described according to the ISCN 2024 guidelines. Spectral karyotyping was performed at Changhua Christian Hospital (Changhua, Taiwan) using the HiSKY probe kit (Applied Spectral Imaging, Carlsbad, CA, USA). Interphase fluorescence in situ hybridization (iFISH) was performed using centromeric probes for chromosome 2, CHR02-10-GR (green); chromosome 9, CHR09-10-RE (red); and chromosome 16, CHR16-10-AQ (aqua) (Empire Genomics, Williamsville, NY, USA). Hybridized nuclei were examined using an Olympus BX63 fluorescence microscope (Olympus, Tokyo, Japan). More than 200 interphase nuclei were captured and analyzed for each sample using GenASIs HiFISH software v8.3.2 (Applied Spectral Imaging). Diploid cells (2n) were defined as those displaying two signals for each probe, whereas polyploid cells (>2n) were defined as those displaying three or more signals for at least one probe.

### 2.7. Cell Cycle Analysis by Flow Cytometry

MV4-11, MV4-11-CR, MOLM-13, and MOLM-13-CR cells were harvested, washed with ice-cold PBS, and fixed in 70% ethanol at −20 °C for at least 24 h. Fixed cells were stained in PBS containing 20 µg/mL propidium iodide (PI), 200 µg/mL RNase A, and 0.1% Triton X-100 for 30 min at room temperature in the dark. Cell-cycle distribution was analyzed using a CytoFLEX flow cytometer (Beckman Coulter, Inc., Brea, CA, USA) and CytExpert software (version 2.4). At least 10,000 events were collected per sample to determine the proportions of cells in the sub-G1, G1, S, and G2/M phases and those with >4N DNA content. The percentage of each population was quantified from the flow cytometry plots and summarized in bar charts.

### 2.8. RNA Sequencing Analysis

Cellular RNA was extracted using the Illustra RNAspin Mini RNA Isolation Kit (Cytiva, Marlborough, MA, USA). RNA concentration, quality, integrity and sequencing were assessed by Taiwan Genomic Industry Alliance Inc. (Taipei, Taiwan) using a Fragment Analyzer system (Agilent Technologies, Santa Clara, CA, USA). Samples with an RNA quality number (RQN) ≥ 8 were used for library preparation. Strand-specific mRNA libraries were prepared using the Illumina Stranded mRNA Prep protocol and sequenced on a NovaSeq X Plus platform (Illumina, San Diego, CA, USA) with 151 bp paired-end reads. Two independent biological replicates per cell line were sequenced, generating >55 million reads per sample. Read quality was assessed using FastQC and MultiQC, followed by adapter and low-quality read removal using Trimmomatic. Clean reads were aligned to the GRCh38 human reference genome using HISAT2. Transcript abundance was estimated using StringTie, and raw gene-level counts were generated using featureCounts. Differential expression was analyzed using DESeq2. Differentially expressed genes (DEGs) were identified based on *p* < 0.05 and log_2_ fold change ≥1 as upregulated, whereas genes with log_2_ fold change ≤−1 were defined as downregulated.

### 2.9. Gene Set Enrichment Analysis (GSEA)

Gene set enrichment analysis (GSEA) was performed using GSEA software (version 4.3.2) with the Hallmark gene set collection from the Molecular Signatures Database (MSigDB) (accessed on 23 June 2026; http://software.broadinstitute.org/gsea/) [26,27]. Gene expression data and sample phenotype labels were analyzed separately for the MV4-11-CR versus MV4-11 and MOLM-13-CR versus MOLM-13 comparisons. Genes were ranked according to the log_2_ ratio of classes. Because each phenotype included two biological replicates, statistical significance was determined using 1000 gene-set permutations. Gene sets with a false discovery rate (FDR) *q*-value < 0.25 and a nominal *p*-value < 0.05 were considered significantly enriched. A positive normalized enrichment score (NES) indicated enrichment in the resistant cells, whereas a negative NES indicated enrichment in the corresponding parental cells. The heatmaps of DEGs were generated using the Heatmapper2 web server (accessed on 6 March 2026; https://server.heatmapper2.ca/) [28].

### 2.10. Transfection of Small Interfering RNA (siRNA) and DCK Expression Plasmid

MV4-11 cells were transfected with either a negative control small interfering RNA (si-Control) or an siRNA targeting human *DCK* (sc-60509; Santa Cruz Biotechnology, Dallas, TX, USA) using the TransIT-X2 Dynamic Delivery System (Mirus Bio, Madison, WI, USA) according to the manufacturer’s instructions. At 24 h after transfection, cells were treated with vehicle or 0.25 μM cytarabine for an additional 48 h. Cell viability was subsequently assessed using the MTT assay.

For *DCK* expression plasmid transfection, MV4-11-CR cells were transfected with either a pCMV6-AC control plasmid or a human *DCK* expression plasmid (pCMV6-AC-*DCK*; OriGene Technologies, Rockville, MD, USA) using the Neon™ NxT Transfection System (Thermo Fisher Scientific), according to the manufacturer’s instructions and as previously described [29]. Electroporation was performed using a two-step pulse program consisting of one pulse at 1350 V for 35 ms, followed by one pulse at 1150 V for 35 ms. After electroporation, cells were cultured in RPMI-1640 medium supplemented with 10% FBS. After 24 h incubation, cells were treated with cytarabine (200 or 400 μM) for 24 h, and cell viability was assessed using the MTT assay.

### 2.11. Compound Combination Treatment and Synergy Analysis

MV4-11, MOLM-13, MV4-11-CR and MOLM-13-CR cells were treated with luvixasertib and midostaurin, either alone or in combination, for 48 h. Cell viability was determined using the MTT assay. Drug interactions were evaluated using CompuSyn software (version 1.0; ComboSyn Inc., Paramus, NJ, USA) according to the median-effect method of Chou and Talalay [30]. Fraction affected (Fa) and combination index (CI) values were calculated for each dose combination. CI values of <1, 1, and >1 indicated synergistic, additive, and antagonistic interactions, respectively.

### 2.12. Statistical Analysis

All experiments were conducted at least three times, and the data were presented as the mean ± standard deviation (SD). Statistical analyses were performed using GraphPad Prism (version 9) (GraphPad Software Inc., San Diego, CA, USA). A Student’s *t*-test was used to compare the two groups. For multiple group comparisons, one-way ANOVA followed by Tukey’s post hoc test was applied. A *p*-value < 0.05 was considered statistically significant.

## 3. Results

### 3.1. Cytarabine-Resistant FLT3-ITD AML Cells Acquire Enhanced Proliferative Capacity Without Uniform FLT3 Activation

**Figure 1:**
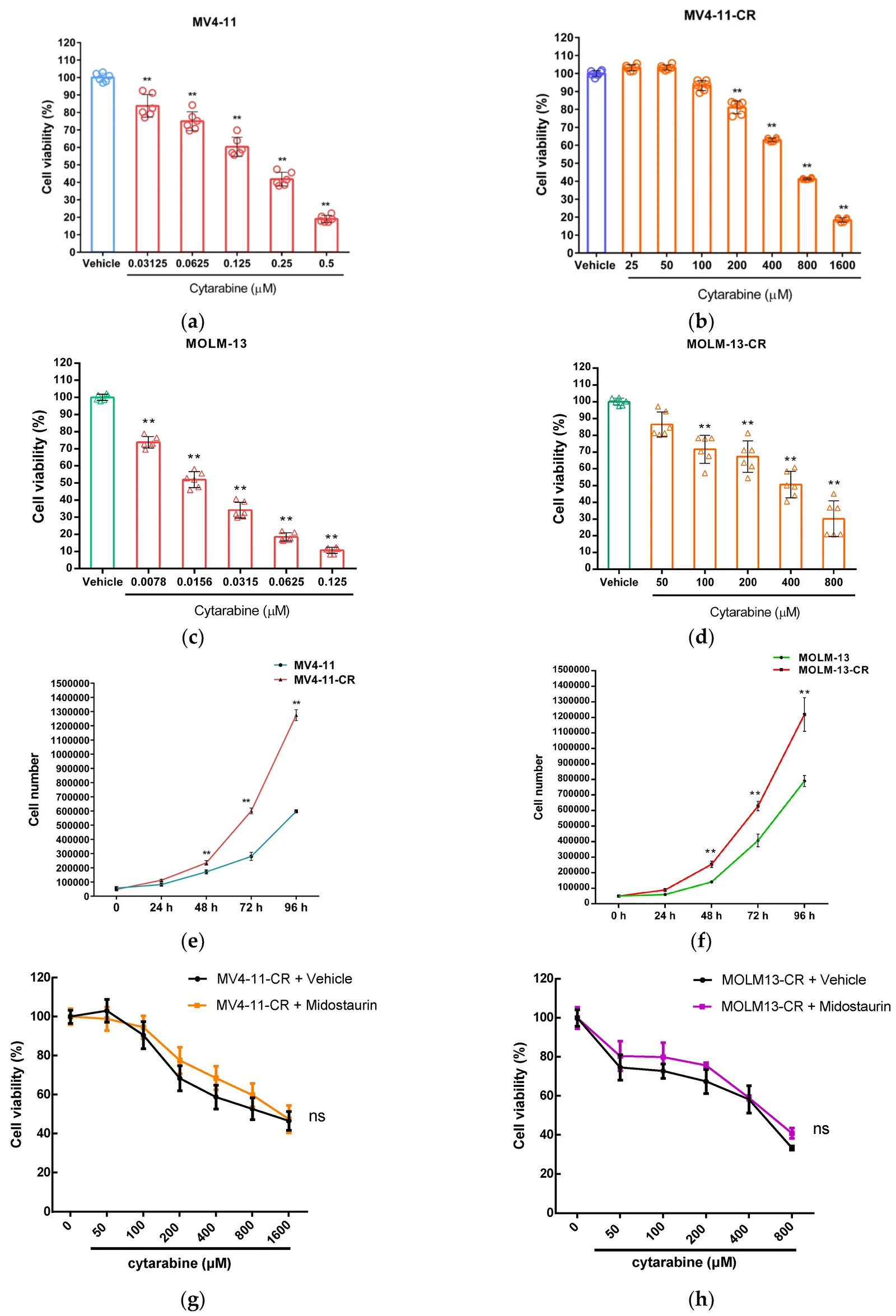
Cytarabine-resistant FLT3-ITD AML cells acquire enhanced proliferative capacity without uniform FLT3 activation. MV4-11, MV4-11-CR, MOLM-13, and MOLM-13-CR cells were treated with increasing concentrations of cytarabine for 48 h, and cell viability was assessed using the MTT assay. (**a**,**b**) Cytarabine dose–response analysis of (**a**) parental MV4-11 and (**b**) MV4-11-CR cells. (**c**,**d**) Cytarabine dose–response analysis of (**c**) parental MOLM-13 and (**d**) MOLM-13-CR cells. Data represent mean ± SD from three independent experiments, with ** *p* < 0.01 indicating significant differences compared to vehicle groups. (**e**,**f**) Proliferation of parental and cytarabine-resistant (**e**) MV4-11 and (**f**) MOLM-13 cells, respectively, assessed by trypan blue exclusion at the indicated time points. Data represent mean ± SD from three independent experiments, with ** *p* < 0.01 indicating significant differences compared to MV4-11 or MOLM-13 cells. (**g**,**h**) Effects of midostaurin pretreatment on cytarabine sensitivity in (**g**) MV4-11-CR and (**h**) MOLM-13-CR cells, respectively. Cells were pretreated with midostaurin (10 nM) for 1 h and subsequently exposed to cytarabine for 48 h. Data are presented as the mean ± SD of at least three independent experiments. ns, not significant.

To establish cellular models of acquired cytarabine resistance, the FLT3-ITD-positive AML cell lines MV4-11 and MOLM-13 were subjected to stepwise cytarabine selection, generating the resistant derivatives MV4-11-CR and MOLM-13-CR, respectively. Cytarabine sensitivity was evaluated after 48 h of treatment. Parental MV4-11 cells exhibited an IC_50_ of 0.17 μM, whereas MV4-11-CR cells displayed an IC_50_ of 591.7 μM, corresponding to an approximately 3480-fold increase in resistance. Similarly, the IC_50_ increased from 0.0177 μM in parental MOLM-13 cells to 368.7 μM in MOLM-13-CR cells, representing an approximately 20,830-fold increase (Figure 1a–d). These findings confirmed the successful establishment of two highly cytarabine-resistant FLT3-ITD AML cell models.

We next examined whether acquired cytarabine resistance was accompanied by altered proliferative capacity. Both MV4-11-CR and MOLM-13-CR cells exhibited significantly greater proliferation than their respective parental counterparts (Figure 1e,f), indicating that the resistant cells had acquired an enhanced growth phenotype.

To determine whether this phenotype was associated with altered FLT3 expression or activation, total and phosphorylated FLT3 protein levels were assessed in parental and resistant cells. In MV4-11-CR cells, both total and phosphorylated FLT3 protein levels were increased compared with parental MV4-11 cells (Figure S1a,b). In contrast, MOLM-13-CR cells showed reduced total and phosphorylated FLT3 protein levels relative to parental MOLM-13 cells (Figure S1c,d). These findings indicate that FLT3 expression and activation were differentially regulated in the two resistant models, suggesting that uniform FLT3 upregulation is not a shared feature of acquired cytarabine resistance.

We further evaluated whether pharmacological FLT3 inhibition could restore cytarabine sensitivity. Parental and resistant cells were pretreated with midostaurin (10 nM) for 1 h, followed by cytarabine exposure for 48 h. Midostaurin pretreatment did not significantly alter cytarabine-induced loss of viability in either resistant cell line (Figure 1g,h; *p* > 0.05). Collectively, these findings demonstrate that cytarabine-resistant FLT3-ITD AML cells acquire enhanced proliferative capacity despite divergent changes in FLT3 expression and activation, suggesting that additional molecular adaptations contribute to the resistant phenotype.

### 3.2. Cytarabine Resistance Is Accompanied by Increased Chromosomal Heterogeneity in FLT3-ITD AML Cells

Chromosomal heterogeneity has been associated with tumor evolution and therapeutic resistance [31,32]. We therefore examined whether acquired cytarabine resistance was accompanied by increased chromosomal abnormalities in FLT3-ITD AML cells.

**Figure 2:**
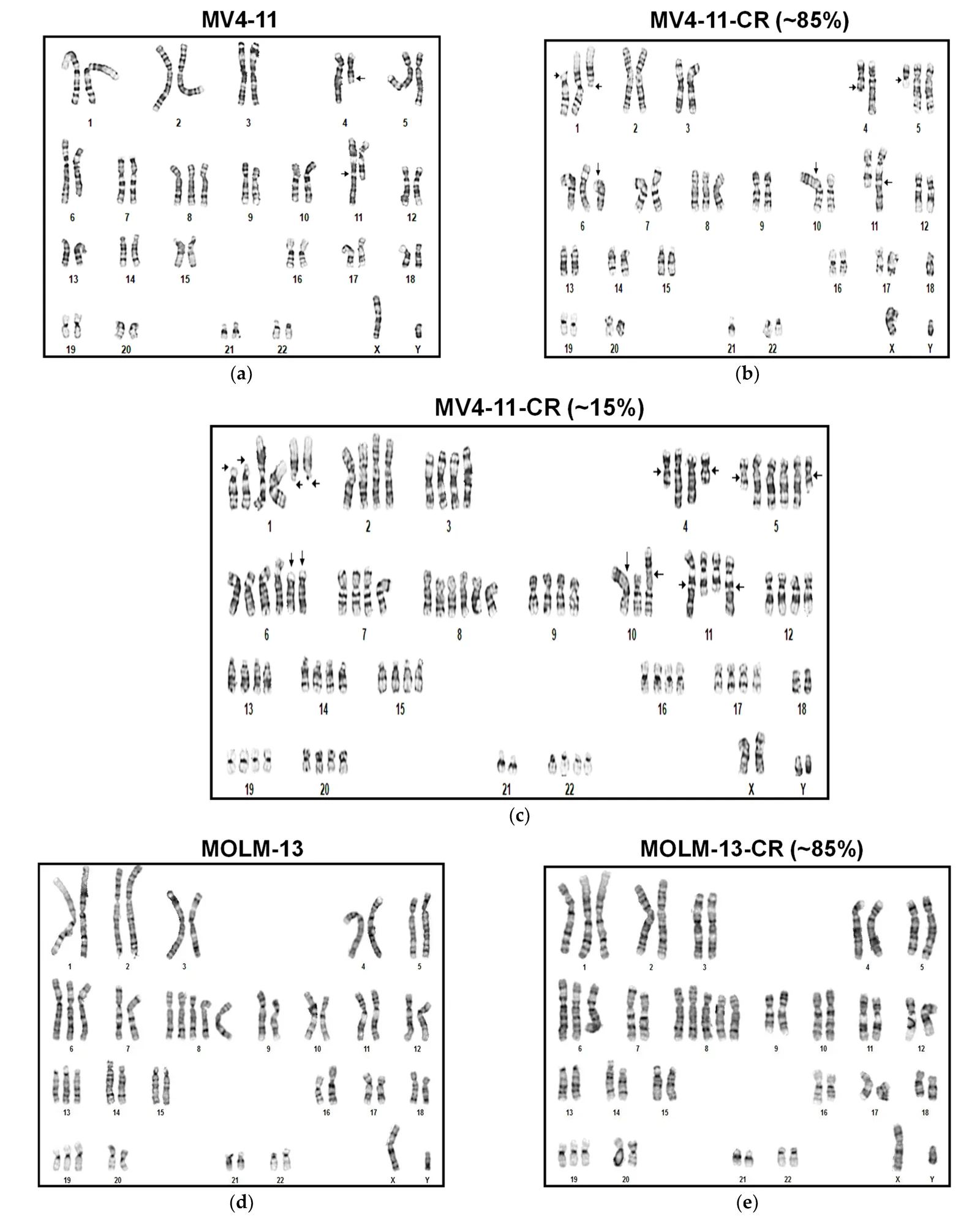

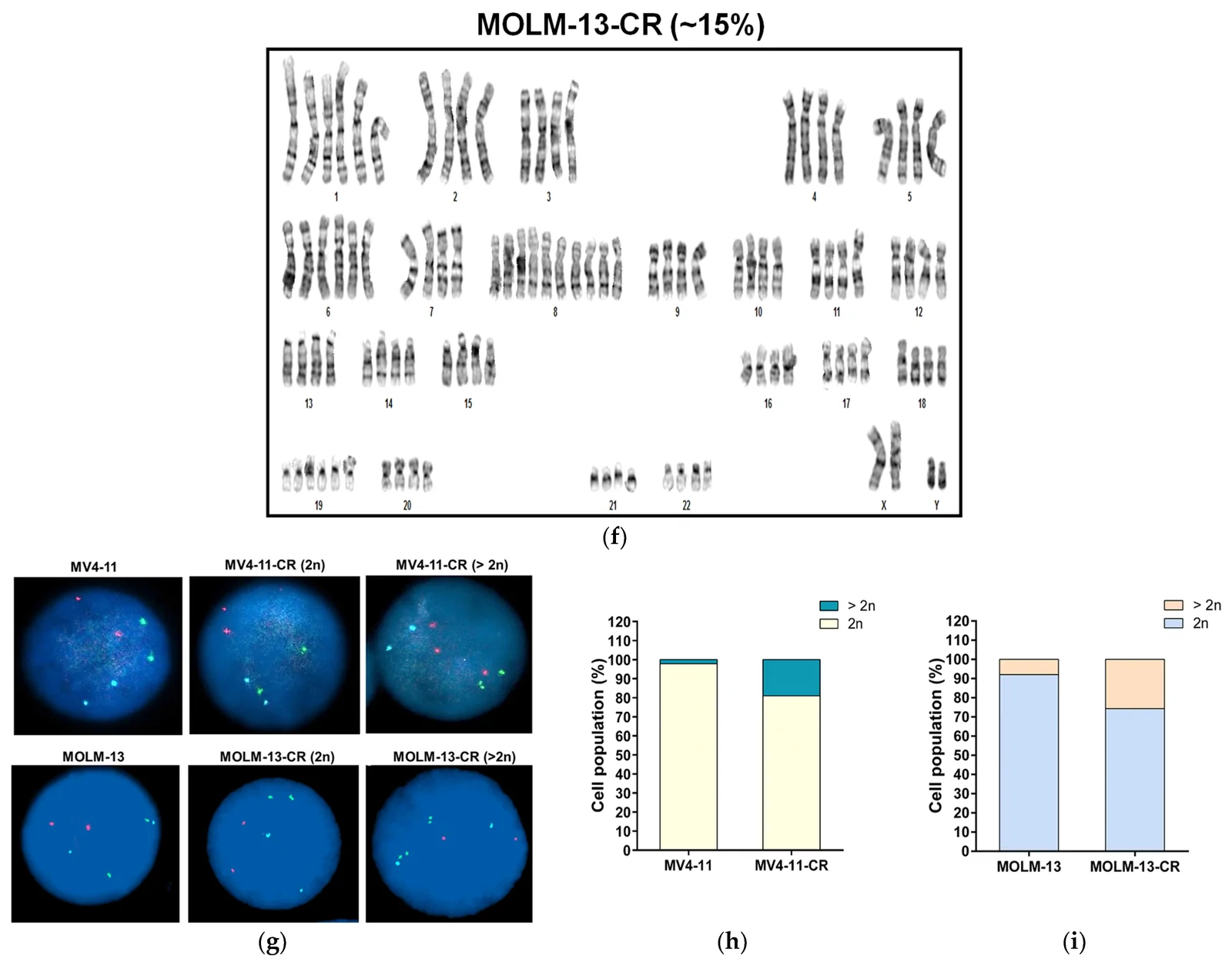
Cytarabine resistance is accompanied by increased chromosomal heterogeneity in FLT3-ITD AML cells. Representative G-banded karyotypes of parental and cytarabine-resistant AML cells are shown. (**a**) Predominant karyotype of parental MV4-11 cells: 47, XY, t(4;11)(q21;q23), +8. (**b**) Near-diploid MV4-11-CR subpopulation (~85%) containing multiple numerical and structural chromosomal abnormalities. (**c**) Near-tetraploid MV4-11-CR subpopulation (~15%) containing 93–95 chromosomes. (**d**) Hyperdiploid karyotype of parental MOLM-13 cells: 52, XY, +6, +8, +8, +8, +13, +19. (**e**) Modified hyperdiploid karyotype of the predominant MOLM-13-CR subpopulation (~85%): 52, XY, +der(1)t(1;6)(q10;p10), +6, +8, +8, +8, +19. (**f**) Hypertetraploid MOLM-13-CR subpopulation (~15%) containing 104 chromosomes. (**g**) Representative interphase FISH images showing ploidy patterns in parental and resistant MV4-11-CR and MOLM-13-CR cells using centromeric probes for chromosome 2 (green), chromosome 9 (red), and chromosome 16 (aqua). More than 200 interphase nuclei were analyzed for each sample. Diploid cells (2n) were defined as those displaying two signals for each probe, whereas polyploid cells (>2n) were defined as those displaying three or more signals for at least one probe. The percentages of diploid and polyploid cells in the parental and resistant MV4-11 and MOLM-13 cells are quantified in panels (**h**) and (**i**), respectively.

G-banding analysis showed that parental MV4-11 cells predominantly exhibited the karyotype 47,XY,t(4;11)(q21;q23),+8 (Figure 2a). In contrast, MV4-11-CR cells displayed increased karyotypic complexity and heterogeneity, comprising two distinct subpopulations. Most cells (~85%) retained a near-diploid chromosome complement of 46–49 chromosomes but harbored multiple structural abnormalities involving chromosomes 1, 4, 5, 6, 10, 11, 18, 21, and Y (Figure 2b). A smaller subpopulation (~15%) exhibited near-tetraploid karyotypes containing 93–95 chromosomes (Figure 2c).

Parental MOLM-13 cells exhibited a hyperdiploid karyotype of 52, XY, +6, +8, +8, +8, +13, +19 (Figure 2d). Most MOLM-13-CR cells (~85%) displayed a modified hyperdiploid karyotype, 52, XY, +der(1)t(1;6)(q10;p10), +6, +8, +8, +8, +19, indicating gain of 1q and 6p material together with loss of the additional chromosome 13 observed in the parental cells (Figure 2e). A minor subpopulation (~15%) exhibited a hyper-tetraploid karyotype containing 104 chromosomes (Figure 2f).

Consistent with the metaphase findings, interphase fluorescence in situ hybridization (iFISH) showed that 97.86% of parental MV4-11 cells exhibited a diploid signal pattern, whereas MV4-11-CR cells comprised diploid and polyploid populations accounting for 81.05% and 18.95% of cells, respectively (Figure 2g,h). Similarly, 92.05% of parental MOLM-13 cells exhibited a diploid signal pattern, whereas MOLM-13-CR cells comprised diploid and polyploid populations accounting for 74.36% and 25.64% of cells, respectively (Figure 2g,i). PI staining was also used to assess baseline cell-cycle distribution. Under untreated conditions, both MV4-11-CR and MOLM-13-CR cells exhibited an increased proportion of cells in the G2/M phase relative to their respective parental cells (Figure S2). MV4-11-CR cells also showed a statistically significant increase in the proportion of cells with >4N DNA content compared with parental MV4-11 cells (5.58% vs. 0.86%) (Figure S2a,b). In contrast, the >4N population was only slightly increased in MOLM-13-CR cells relative to parental MOLM-13 cells (0.50% vs. 0.24%), and the difference was not statistically significant (Figure S2c,d). These findings indicate altered baseline cell-cycle distributions in both resistant models, with a significant increase in cells with >4N DNA content detected only in MV4-11-CR cells.

### 3.3. Transcriptomic Profiling Identifies Shared Resistance-Associated Alterations and DCK Downregulation

**Figure 3:**
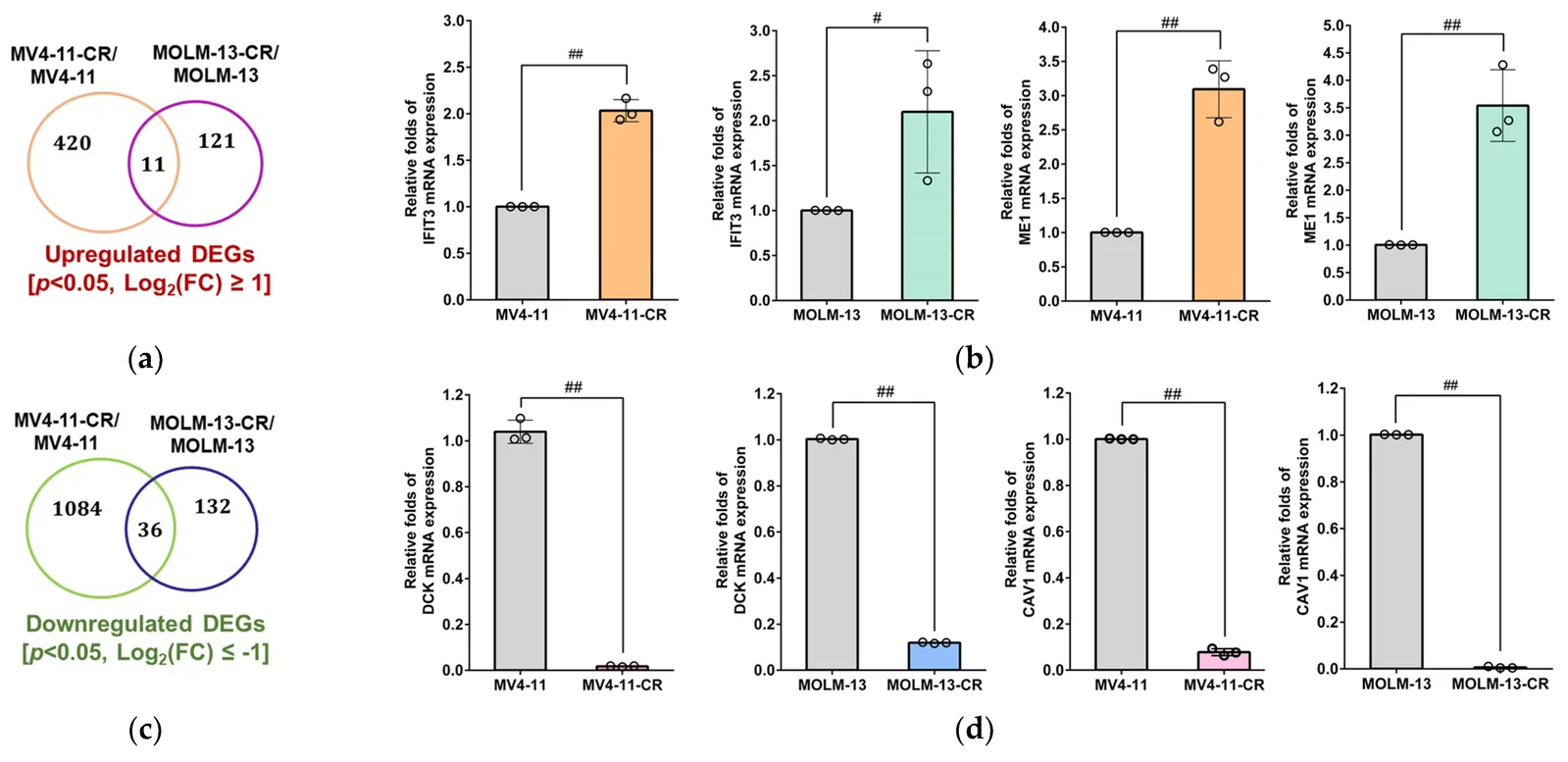
Differential gene expression and RT-qPCR validation. RNA sequencing was performed to identify DEGs in cytarabine-resistant AML cells. (**a**) Number of genes significantly upregulated in MV4-11-CR and MOLM-13-CR cells relative to their parental counterparts (*p* < 0.05, log_2_ fold change ≥ 1). (**b**) RT-qPCR validation of *IFIT3* and *ME1* expression. (**c**) Number of genes significantly downregulated in MV4-11-CR and MOLM-13-CR cells relative to their parental counterparts (*p* < 0.05, log_2_ fold change ≤ −1. (**d**) RT-qPCR validation of *DCK* and *CAV1* expression. RT-qPCR data are presented as mean ± SD from at least three independent experiments. ^#^ *p* < 0.05 and ^##^ *p* < 0.01 indicate significant differences compared to the respective parental MV4-11 or MOLM-13 control groups.

To characterize the molecular alterations associated with acquired cytarabine resistance, we performed RNA sequencing of parental and resistant FLT3-ITD AML cells. Using thresholds of *p* < 0.05 and log_2_ fold change (FC) ≥ 1, we identified 431 and 132 upregulated differentially expressed genes (DEGs) in MV4-11-CR and MOLM-13-CR cells, respectively, relative to their parental counterparts (Figure 3a). Eleven genes were commonly upregulated in both resistant cell lines (Figure S3a,b). Among these, the increased expression of *IFIT3* and *ME1* was further confirmed by RT-qPCR (Figure 3b).

Using thresholds of *p* < 0.05 and log_2_ FC ≤ −1, we identified 1120 and 168 downregulated DEGs in MV4-11-CR and MOLM-13-CR cells, respectively (Figure 3c). Thirty-six genes were commonly downregulated in both resistant models (Figure S3c,d). RT-qPCR validated significantly reduced expression of *DCK* and *CAV1* in both resistant cell lines compared with their respective parental counterparts (Figure 3d).

**Figure 4:**
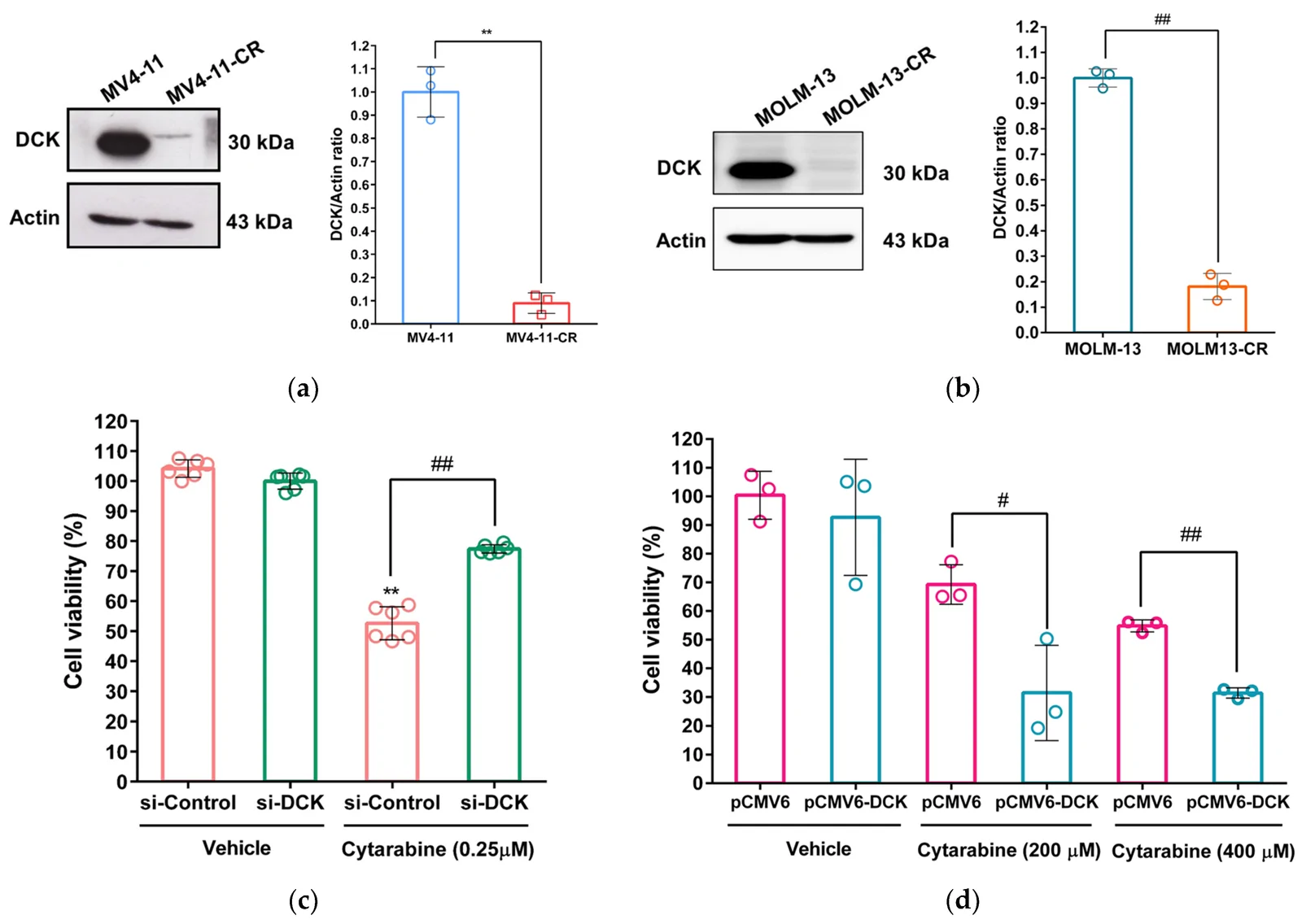
Downregulation of *DCK* expression attenuates the cytotoxic response to cytarabine. Western blot analysis and quantification of *DCK* protein expression in (**a**) MV4-11 and MV4-11-CR cells and (**b**) MOLM-13 and MOLM-13-CR cells. *DCK* protein levels were normalized to actin. Data are presented as the mean ± SD from at least three independent experiments. ** *p* < 0.01 and ^##^ *p* < 0.01 indicate significant differences compared with the MV4-11 and MOLM-13 control groups, respectively. (**c**) Effect of *DCK* knockdown on cytarabine sensitivity in parental MV4-11 cells. Twenty-four hours after transfection with control siRNA (si-Control) or *DCK*-targeting siRNA (si-*DCK*), cells were treated with vehicle or cytarabine (0.25 μM) for an additional 48 h, followed by an MTT assay. Data are presented as the mean ± SD from three independent experiments. ** *p* < 0.01 indicates a significant difference compared with the corresponding vehicle-treated group, whereas ^##^ *p* < 0.01 indicates a significant difference compared with the si-Control-transfected group. (**d**) MV4-11-CR cells were transfected with either a pCMV6-AC control plasmid (pCMV6) or a pCMV6-AC-*DCK* expression plasmid (pCMV6-*DCK*) by electroporation. After 24 h, cells were treated with vehicle or cytarabine (200 and 400 μM) for 24 h. Cell viability was determined using the MTT assay, with that of vehicle-treated pCMV6 control-transfected cells defined as 100%. Data are presented as the mean ± SD from three independent experiments. ^#^ *p* < 0.05 and ^##^ *p* < 0.01 indicate significant differences compared with the corresponding pCMV6 control-transfected cells.

Because *DCK* catalyzes the initial phosphorylation of cytarabine, a critical step in its intracellular activation [33], we further investigated its contribution to the resistant phenotype. Consistent with the RNA-sequencing and RT-qPCR results, western blot analysis confirmed that *DCK* protein expression was significantly reduced in both cytarabine-resistant cell lines compared with their parental counterparts (Figure 4a,b). To determine the functional significance of *DCK* downregulation, parental MV4-11 cells were transfected with *DCK*-targeting small interfering RNA (si-*DCK*) and subsequently treated with cytarabine. *DCK* knockdown increased cell viability by approximately 25% compared with control siRNA-transfected cells following cytarabine treatment (Figure 4c). Furthermore, we examined the effect of *DCK* re-expression in MV4-11-CR cells. As shown in Figure 4d, transfection with the *DCK* expression plasmid partially restored sensitivity to cytarabine (200 and 400 μM) in MV4-11-CR cells. These findings support the notion that reduced *DCK* expression attenuates the cytotoxic effects of cytarabine and contributes, at least in part, to acquired cytarabine resistance in FLT3-ITD AML cells.

### 3.4. Mitotic Regulatory Programs Are Enriched in Cytarabine-Resistant AML Cells

**Figure 5:**
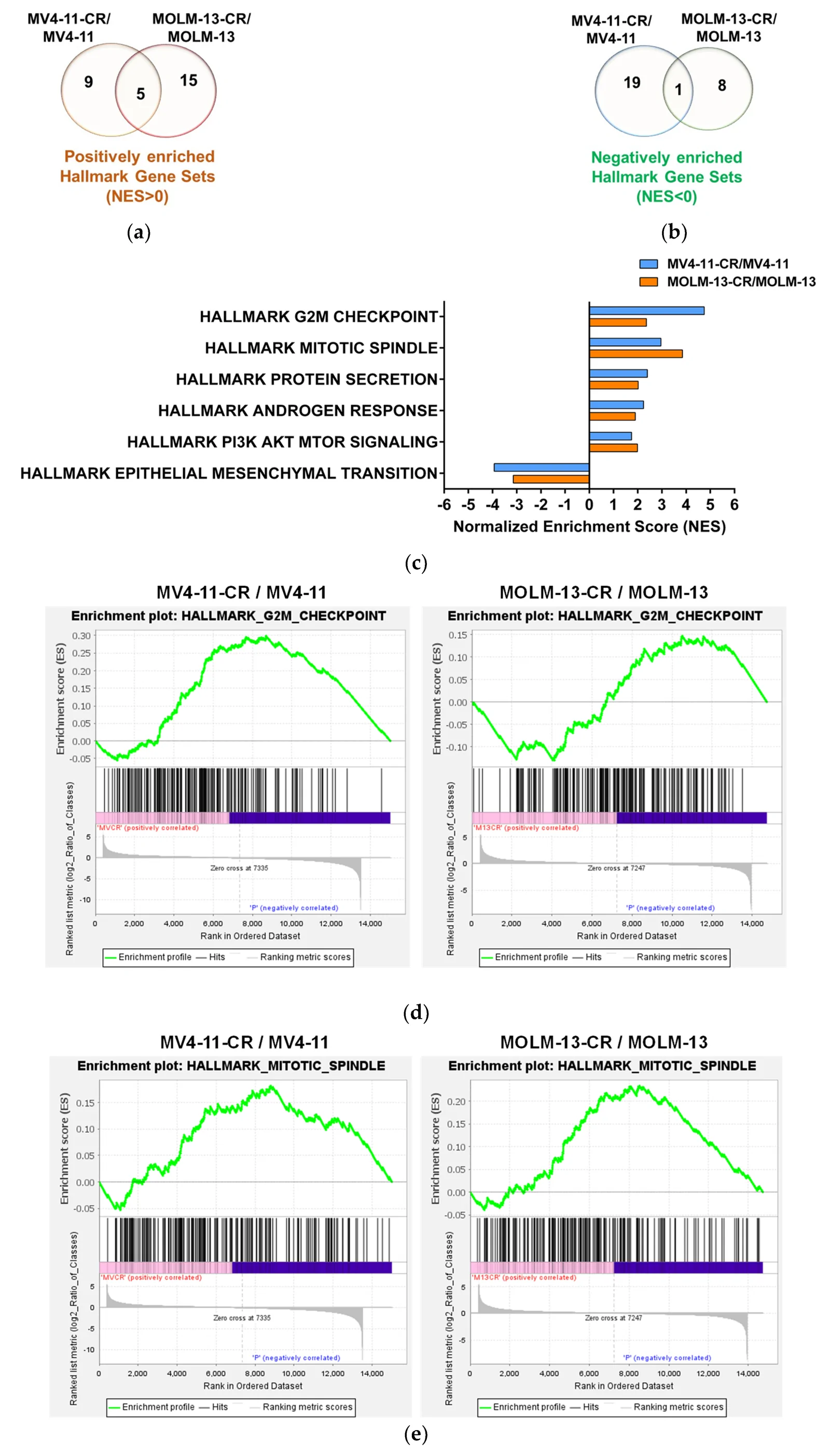
Mitotic regulatory programs are enriched in cytarabine-resistant AML cells. GSEA was performed using the Hallmark gene set collection from the Molecular Signatures Database (MSigDB). Comparisons were performed separately between MV4-11-CR and MV4-11 cells and between MOLM-13-CR and MOLM-13 cells. (**a**) Hallmark gene sets significantly positively enriched in cytarabine-resistant cells. (**b**) Hallmark gene sets significantly negatively enriched in cytarabine-resistant cells. (**c**) Overlap of positively and negatively enriched Hallmark gene sets between the two resistant models. Five positively and one negatively enriched gene set were shared by MV4-11-CR and MOLM-13-CR cells. GSEA enrichment plots for (**d**) the Hallmark G2/M Checkpoint gene set and (**e**) the Hallmark Mitotic Spindle gene set in MV4-11-CR and MOLM-13-CR cells. Gene sets with a false discovery rate (FDR) *q* value < 0.25 and a nominal *p* value < 0.05 were considered significantly enriched. NES, normalized enrichment score; FDR, false discovery rate.

To characterize the biological pathways associated with acquired cytarabine resistance, gene expression profiles of MV4-11-CR and MOLM-13-CR cells were compared with those of their respective parental counterparts using GSEA with the Hallmark gene set collection. As shown in Tables S2 and S3 Hallmark gene sets were significantly positively enriched in MV4-11-CR and MOLM-13-CR cells, respectively. Five gene sets were commonly positively enriched in both resistant models including G2/M checkpoint, mitotic spindle, protein secretion, androgen response, and PI3K-AKT-mTOR signaling (Figure 5a,c). Conversely, 20 and 9 Hallmark gene sets were significantly negatively enriched in MV4-11-CR and MOLM-13-CR cells, respectively (Tables S4 and S5). Epithelial-mesenchymal transition was the only negatively enriched gene set shared between the two resistant models (Figure 5b,c).

Notably, the G2/M checkpoint and mitotic spindle gene sets were among the most prominently positively enriched programs in both MV4-11-CR and MOLM-13-CR cells (Figure 5d,e). Because these pathways are central to mitotic progression and chromosome segregation fidelity [34,35,36], their shared enrichment provided a rationale for investigating whether mitotic checkpoint regulation represents a therapeutically exploitable vulnerability in cytarabine-resistant AML cells.

### 3.5. Effects of Combined TTK/Mps1 and FLT3 Inhibition in Cytarabine-Resistant AML Cells

We next investigated whether pharmacological disruption of mitotic checkpoint control could enhance the antileukemic activity of FLT3 inhibition in cytarabine-resistant AML cells. Luvixasertib (CFI-402257) is a selective inhibitor of the spindle assembly checkpoint kinase TTK/Mps1 that disrupts chromosome alignment and mitotic checkpoint function, thereby promoting mitotic failure and cell death [37].

**Figure 6:**
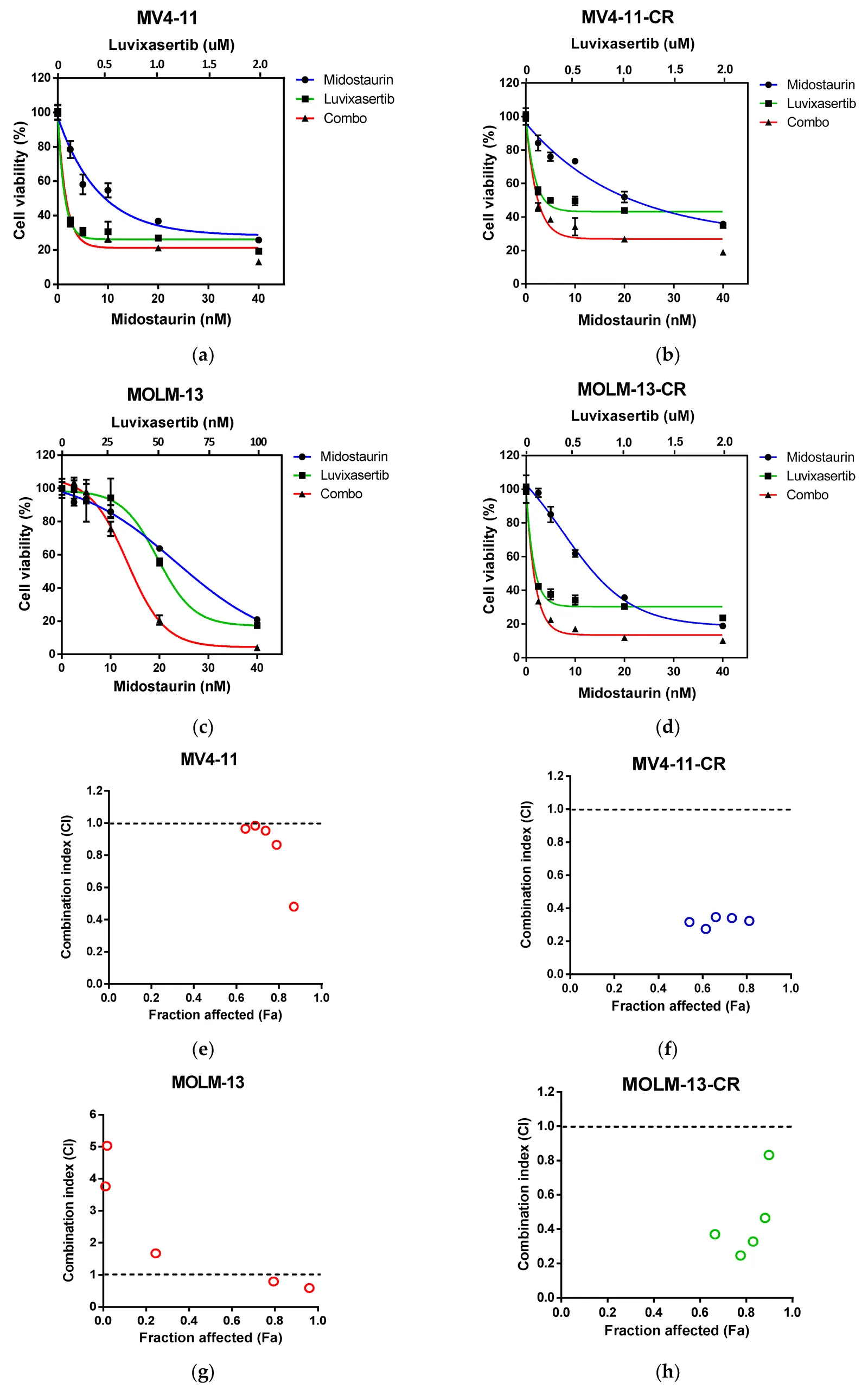
Combined inhibition of TTK/Mps1 and FLT3 synergistically suppresses cell growth in cytarabine-resistant AML cells. MV4-11-CR and MOLM-13-CR cells were treated with increasing concentrations of luvixasertib and midostaurin, either alone or in combination (combo), for 48 h, followed by assessment of cell viability using the MTT assay. (**a**–**d**) Dose–response curves for MV4-11, MV4-11-CR, MOLM-13 and MOLM-13-CR cells, respectively. (**e**–**h**) Fraction affected–combination index (Fa-CI) plots for MV4-11, MV4-11-CR, MOLM-13 and MOLM-13-CR cells, respectively. Drug interactions were evaluated using the Chou–Talalay median-effect method implemented in CompuSyn software (Version 1.0). CI values <1, =1, and >1 indicate synergistic, additive, and antagonistic interactions, respectively. The dashed horizontal line indicates CI = 1. Fa, fraction affected; CI, combination index.

We first examined *TTK* mRNA expression in parental and cytarabine-resistant AML cells. As shown in Figure S4, *TTK* mRNA expression was markedly higher in MV4-11-CR cells than in parental MV4-11 cells, whereas a small but statistically significant increase (approximately 1.2-fold) was observed in MOLM-13-CR cells relative to parental MOLM-13 cells. Furthermore, to compare the effects of TTK/Mps1 inhibition, parental and cytarabine-resistant AML cells were treated with luvixasertib and midostaurin, alone or in combination, for 48 h. The combination reduced cell viability more effectively than either agent alone (Figure 6a–d). CompuSyn analysis revealed distinct interaction profiles, with more consistent synergistic interactions in resistant cells. MV4-11-CR cells showed synergy across all tested doses (Fa = 0.540–0.811; CI = 0.275–0.347), whereas parental MV4-11 cells showed weak synergy or near-additive effects at four doses (CI = 0.865–0.985) and synergy at the highest dose (CI = 0.481; Figure 6e,f). MOLM-13-CR cells also showed synergy across all doses (Fa = 0.665–0.898; CI = 0.246–0.833), whereas parental MOLM-13 cells showed antagonism at lower effect levels and synergy at higher effect levels (CI = 0.591–5.035; Figure 6g,h). Collectively, these findings indicate that combined TTK/Mps1 and FLT3 inhibition synergistically reduces the viability of cytarabine-resistant FLT3-ITD AML cells. Although combination effects were also observed in parental cells, the interaction profiles differed between parental and resistant models. These results support further evaluation of this combination as a potential therapeutic strategy for cytarabine-resistant AML.

## 4. Discussion

Cytarabine resistance remains a major obstacle in the treatment of AML [38,39]. In this study, we established the cytarabine-resistant cell models MV4-11-CR and MOLM-13-CR and identified several features associated with the resistant phenotype, including heterogeneous FLT3 regulation, reduced *DCK* expression, increased numerical and structural chromosomal heterogeneity, and enrichment of mitotic regulatory programs. Moreover, combined inhibition of TTK/Mps1 and FLT3 produced synergistic antileukemic effects in both resistant cell lines, suggesting that mitotic checkpoint regulation may represent a therapeutically exploitable vulnerability in cytarabine-resistant FLT3-ITD AML.

FLT3 mutations occur in approximately 30% of AML cases, with FLT3-ITD being the most common subtype and associated with a high disease burden, frequent relapse, and adverse clinical outcomes [11]. However, the two resistant models exhibited distinct patterns of FLT3 regulation. MV4-11-CR cells showed increased FLT3 expression and phosphorylation together with enhanced proliferation, whereas FLT3 expression and phosphorylation were reduced in MOLM-13-CR cells. These contrasting findings indicate that acquired cytarabine resistance does not uniformly depend on enhanced FLT3 signaling, even among FLT3-ITD AML cells. Previous studies have shown that FLT3-mutant AML cells can maintain survival through FLT3-independent or compensatory mechanisms [11,40]. Accordingly, the divergent FLT3 profiles observed here suggest that additional molecular adaptations contribute to the proliferative and drug-resistant phenotypes of cytarabine-resistant FLT3-ITD AML cells.

Chromosome heterogeneity and instability could promote tumor evolution by generating numerical and structural chromosomal diversity and has been associated with poor prognosis and therapeutic resistance [41,42]. Both cytarabine-resistant cell lines exhibited greater karyotypic complexity than their parental counterparts. MV4-11-CR cells acquired multiple structural abnormalities and contained both near-diploid and near-tetraploid subpopulations. MOLM-13-CR cells similarly displayed a modified hyper-diploid karyotype characterized by gain of 1q and 6p material, loss of the additional chromosome 13 present in the parental cells, and the emergence of a hyper-tetraploid subpopulation. Interphase FISH further confirmed increased proportions of polyploid cells in both resistant models. Together, these observations indicate that acquired cytarabine resistance is accompanied by increased chromosomal heterogeneity and the expansion of polyploid subpopulations.

The emergence of polyploid subpopulations may be relevant to therapeutic adaptation, as polyploid cancer cells have been associated with increased tumor heterogeneity, treatment resistance, and disease recurrence [43]. However, the present cytogenetic analyses primarily demonstrate increased chromosomal heterogeneity rather than directly measuring ongoing chromosome mis-segregation. Longitudinal single-cell analyses, live-cell imaging, and direct assessment of mitotic errors will therefore be required to define the dynamics of chromosome instability and determine whether specific polyploid subpopulations preferentially contribute to the cytarabine-resistant phenotype.

FLT3-ITD signaling has previously been linked to reactive oxygen species production, DNA damage, and genomic instability through STAT5- and RAC1-dependent mechanisms [44,45,46]. Nevertheless, the divergent FLT3 expression patterns observed in MV4-11-CR and MOLM-13-CR cells suggest that the increased chromosomal heterogeneity in the resistant models cannot be attributed solely to enhanced FLT3 activity. Chromosomal remodeling may instead arise from broader adaptations to prolonged cytarabine exposure, including altered DNA damage responses, mitotic checkpoint dysregulation, or the selection of pre-existing heterogeneous subclones with distinct chromosomal compositions [47]. However, the temporal and causal relationships among FLT3 signaling, CIN, and cytarabine resistance remain to be clarified.

Transcriptomic analysis further indicated changes in mitosis-related gene expression associated with the resistant phenotype. The Hallmark G2/M Checkpoint and Mitotic Spindle gene sets were among the most prominently enriched gene sets in both resistant cell lines. This shared enrichment suggests coordinated transcriptional changes in genes involved in chromosome alignment, spindle attachment, and mitotic progression. Among the mitosis-related genes, *TTK*, which encodes the Mps1 kinase, was prioritized for further investigation based not solely on the magnitude of its expression change but also on its central role in spindle assembly checkpoint control and its pharmacological tractability. RT-qPCR analysis showed that *TTK* mRNA expression was markedly increased in MV4-11-CR cells and showed a small but statistically significant increase in MOLM-13-CR cells relative to their respective parental cells. Mps1 delays anaphase onset until chromosomes are properly attached to the mitotic spindle [18]. Cancer cells harboring chromosome segregation defects may therefore become increasingly dependent on this checkpoint to maintain sufficient mitotic fidelity for continued survival [19]. Although these findings identify TTK/Mps1 as a candidate therapeutic vulnerability, direct dependence on TTK/Mps1 has not yet been established. Further studies involving genetic inhibition of *TTK* and pharmacological targeting of other mitotic regulators, such as KIF11, AURKA, and AURKB, are needed to determine whether this vulnerability extends to other components of the mitotic regulatory network.

Acquired cytarabine resistance was also associated with altered baseline cell-cycle distribution. Under untreated conditions, both MV4-11-CR and MOLM-13-CR cells exhibited an increased proportion of cells in the G2/M phase relative to their respective parental cells, indicating that acquired cytarabine resistance is associated with altered baseline cell cycle regulation. A significant increase in cells with >4N DNA content was observed only in MV4-11-CR cells, whereas MOLM-13-CR cells showed only a slight, nonsignificant increase. These findings indicate altered cell cycle distributions in both resistant models, although changes in the >4N population were not consistent between the two models. Together with the enrichment of G2/M checkpoint and mitotic spindle gene sets, these findings further support altered cell-cycle and mitotic regulation in the resistant phenotype. However, because cell-cycle responses to acute cytarabine treatment were not directly examined, whether the resistant cells exhibit altered cytarabine-induced cell-cycle arrest remains unclear. Differentiation responses were also not assessed and warrant further investigation.

Consistent with this possibility, the TTK/Mps1 inhibitor luvixasertib enhanced the activity of midostaurin in MV4-11-CR and MOLM-13-CR cells. CompuSyn analysis showed synergistic interactions across all tested dose combinations in both resistant models. In the corresponding parental cells, MV4-11 cells showed weak synergy or near-additive effects at most doses, whereas MOLM-13 cells displayed antagonism at lower effect levels and synergy at higher effect levels. Thus, the combination effect was not restricted to cytarabine-resistant cells but was more consistently synergistic in the resistant models. Combined targeting of TTK/Mps1 and FLT3 signaling may therefore warrant further investigation as a potential strategy for cytarabine-resistant AML.

Reduced *DCK* expression represented another shared feature of acquired cytarabine resistance. Cytarabine enters leukemia cells primarily through nucleoside transporters and requires phosphorylation by *DCK* to generate its active triphosphate metabolite [7]. Reduced *DCK* expression or activity limits cytarabine activation and is a well-established mechanism of resistance [48,49]. In the present study, *DCK* mRNA and *DCK* protein levels were reduced in both resistant cell lines. Moreover, *DCK* knockdown increased the viability of parental MV4-11 cells following cytarabine treatment, whereas *DCK* re-expression in MV4-11-CR cells partially restored cytarabine sensitivity. These findings support a functional contribution of *DCK* downregulation to the resistant phenotype.

In MV4-11-CR cells, one structurally altered chromosome 4 appeared to lack the region containing *DCK*, located at chromosome 4q13.3–q21.1, raising the possibility of loss of one *DCK* allele [50]. However, this structural alteration alone is unlikely to fully account for the marked reduction in *DCK* expression, particularly because *DCK* downregulation was also observed in MOLM-13-CR cells. Additional transcriptional or epigenetic mechanisms may therefore be involved. DNA methylation, histone modifications, and altered transcription factor activity can suppress gene expression without altering the underlying DNA sequence [51]. Further studies are needed to determine whether such mechanisms contribute to *DCK* repression in cytarabine-resistant AML.

The resistant cell lines also exhibited broader transcriptional adaptations involving cell-cycle regulation, DNA replication and repair, metabolism, and stress responses. Importantly, the overlap between the two models was incomplete, indicating that acquired resistance arises through both shared and cell-line-specific mechanisms. *DCK* downregulation and enrichment of mitotic pathways were common features, whereas FLT3 regulation differed substantially between the two cell lines. This heterogeneity highlights the limitations of attributing cytarabine resistance to a single dominant pathway and supports the use of complementary cellular models when investigating resistance mechanisms.

Public AML cohorts contain extensive molecular and clinical data, but no sufficiently large cohort clearly distinguishes cytarabine-sensitive from cytarabine-resistant AML while providing expression or functional data on *DCK*, mitotic spindle regulators, and CIN-related features. In datasets with relevant treatment-response information, small sample sizes and the use of multidrug regimens precluded robust cytarabine-specific comparisons. Moreover, relapsed or refractory disease is not equivalent to cytarabine resistance. Nevertheless, higher *DCK* expression and *DCK* enzymatic activity have been associated with favorable treatment responses and longer event-free survival in patients receiving cytarabine-containing regimens [8,49], supporting the clinical relevance of *DCK*-mediated cytarabine activation. Patient-level relationships among mitotic regulatory changes, chromosomal heterogeneity, and acquired cytarabine resistance remain poorly defined and require validation in larger longitudinal cohorts with cytarabine-specific response data.

Several limitations should be acknowledged. First, this study was based on two established FLT3-ITD AML cell lines and may not fully reproduce the genetic diversity, clonal architecture, or microenvironmental influences present in patients. Validation in primary AML samples, patient-derived models, and in vivo systems will therefore be necessary. Second, although increased chromosomal heterogeneity and polyploid subpopulations were consistently detected, the mechanisms generating these abnormalities and their direct contribution to the cytarabine-resistant phenotype remain unresolved. Third, although combined luvixasertib and midostaurin treatment showed more consistent synergistic interactions in the resistant models than in the corresponding parental cells, the combination also exerted activity in parental cells. Therefore, its resistance-associated selectivity, therapeutic window, and efficacy require further evaluation in preclinical models. In addition, the functional analyses in the present study were primarily based on short-term cell-viability and proliferation assays. Future studies incorporating colony-forming assays and leukemia stem/progenitor models will be important to determine whether combined TTK/Mps1 and FLT3 inhibition also affects long-term clonogenic potential and stem/progenitor properties. Finally, the mechanisms responsible for *DCK* downregulation remain to be clarified.

## 5. Conclusions

Our study highlights the complex molecular mechanisms underlying cytarabine resistance in AML. Acquired cytarabine resistance in FLT3-ITD AML is associated with divergent FLT3 regulation, reduced *DCK* expression, increased chromosomal heterogeneity, and enrichment of mitotic regulatory programs. The shared enrichment of the G2/M checkpoint and mitotic spindle pathways, together with the synergistic effects of combined TTK/Mps1 and FLT3 inhibition, supports mitotic checkpoint regulation as a potential therapeutic vulnerability in cytarabine-resistant AML cells. These findings underscore the multifactorial nature of cytarabine resistance and support further investigation of strategies aimed at restoring *DCK*-mediated cytarabine activation and simultaneously targeting TTK/Mps1 and FLT3 signaling in cytarabine-resistant FLT3-ITD AML.

## Data Availability

The original contributions presented in this study are included in the article/Supplementary Materials. Further inquiries can be directed to the corresponding author.

## Supplementary Materials

The following supporting information can be downloaded at: https://www.mdpi.com/article/10.3390/cimb48090966/s1.
