# Supplementary material for: Chromosomal Heterogeneity and Enrichment of Mitotic Regulatory Programs Reveal a Therapeutic Vulnerability in Cytarabine-Resistant FLT3-ITD AML
Source: Curr Issues Mol Biol. 2026 Sep 21;48(9):966. doi: 10.3390/cimb48090966 (PMC13605166; doi:10.3390/cimb48090966)
Supplement: Supplementary file 1 [file cimb-48-00966-s001.zip › cimb-4517415-supplementary.pdf]

Table S1. The primer pairs used in RT-qPCR

Genes	Primers
Hu-IFIT3	F: 5'-GAACATGCTGACCAAGCAGA-3'
	R: 5'-CAGTTGTGTCCACCCTTCCT-3'
Hu-ME1	F: 5'-AGTTTGGTGTTTCGGAAGCC-3'
	R: 5'-CTCCCAAGCCAAGAATACGC-3'
Hu-DCK	F: 5'-CGTCTTTCTCAGCCAGCTCT-3'
	R: 5'-TCAGGAACCACTTCCCAATC-3'
Hu-CAV1	F: 5'-GAGCTGAGCAGAAGCAAGT-3'
	R: 5'-TCCCTTCTGGTTCTGCAATC-3'
Hu-TTK	F: 5'-ATTTTCCGGTTCACTTGGGC-3'
	R: 5'-TGGTGGCATGTTCTCTCCAT-3'
Hu-β-actin	F: 5'-CATGTACGTTGCTATCCAGGC-3'
	R: 5'-CTCCTTAATGTCACGCACGAT-3'

Table S2. Hallmark gene sets positively enriched in MV4-11-CR cells

HALLMARK	Size	ES	NES	NOM p-value	FDR q-value	Leading edge
HALLMARK_MYC_TARGETS_V1	200	0.36	5.99	0	0	tags=93%, list=57%, signal=213%
HALLMARK_E2F_TARGETS	200	0.29	4.76	0	0	tags=85%, list=56%, signal=192%
HALLMARK_G2M_CHECKPOINT	197	0.3	4.75	0	0	tags=87%, list=58%, signal=204%
HALLMARK_OXIDATIVE_PHOSPHORYLATION	198	0.25	4.07	0	0	tags=90%, list=65%, signal=257%
HALLMARK_MYC_TARGETS_V2	58	0.38	3.42	0	0	tags=88%, list=50%, signal=174%
HALLMARK_MTORC1_SIGNALING	197	0.2	3.18	0	0	tags=66%, list=47%, signal=124%
HALLMARK_DNA_REPAIR	148	0.22	3.08	0	0	tags=95%, list=73%, signal=348%
HALLMARK_UNFOLDED_PROTEIN_RESPONSE	112	0.25	3.04	0	0	tags=77%, list=52%, signal=160%
HALLMARK_MITOTIC_SPINDLE	196	0.18	2.96	0	0	tags=77%, list=59%, signal=182%
HALLMARK_PROTEIN_SECRETION	93	0.21	2.4	0	0.001	tags=63%, list=43%, signal=111%
HALLMARK_ANDROGEN_RESPONSE	86	0.21	2.24	0	0.002	tags=69%, list=48%, signal=131%
HALLMARK_PI3K_AKT_MTOR_SIGNALING	98	0.15	1.75	0.02	0.035	tags=78%, list=62%, signal=205%
HALLMARK_UV_RESPONSE_UP	143	0.11	1.59	0.043	0.071	tags=87%, list=76%, signal=352%
HALLMARK_SPERMATOGENESIS	98	0.14	1.58	0.034	0.068	tags=54%, list=41%, signal=91%

Table S3. Hallmark gene sets positively enriched in MOLM-13-CR cells

HALLMARK	Size	ES	NES	NOM p-value	FDR q-value	Leading edge
HALLMARK_MITOTIC_SPINDLE	194	0.23	3.85	0	0	tags=81%, list=58%, signal=189%
HALLMARK_INTERFERON_GAMMA_RESPONSE	183	0.21	3.29	0	0	tags=57%, list=37%, signal=90%
HALLMARK_IL2_STAT5_SIGNALING	178	0.19	3.04	0	0	tags=47%, list=28%, signal=65%
HALLMARK_INFLAMMATORY_RESPONSE	173	0.2	3	0	0	tags=49%, list=29%, signal=68%
HALLMARK_INTERFERON_ALPHA_RESPONSE	93	0.26	2.95	0	0	tags=56%, list=30%, signal=79%
HALLMARK_KRAS_SIGNALING_DN	118	0.19	2.48	0	0	tags=34%, list=15%, signal=39%
HALLMARK_HEME_METABOLISM	170	0.16	2.41	0	0.001	tags=62%, list=47%, signal=115%
HALLMARK_TGF_BETA_SIGNALING	51	0.29	2.38	0	0.001	tags=88%, list=60%, signal=218%
HALLMARK_APOPTOSIS	147	0.17	2.37	0	0.001	tags=46%, list=29%, signal=63%
HALLMARK_G2M_CHECKPOINT	197	0.15	2.36	0	0.001	tags=86%, list=71%, signal=295%
HALLMARK_KRAS_SIGNALING_UP	147	0.16	2.32	0	0.001	tags=44%, list=28%, signal=60%
HALLMARK_IL6_JAK_STAT3_SIGNALING	75	0.22	2.31	0	0.001	tags=63%, list=40%, signal=104%
HALLMARK_MYOGENESIS	165	0.15	2.25	0.002	0.002	tags=33%, list=18%, signal=40%
HALLMARK_PROTEIN_SECRETION	93	0.18	2.02	0.004	0.013	tags=74%, list=56%, signal=168%

HALLMARK_PI3K_AKT_MTOR_SIGNALING	98	0.17	1.99	0.006	0.014	tags=68%, list=52%, signal=140%
HALLMARK_COAGULATION	104	0.17	1.98	0.006	0.014	tags=38%, list=21%, signal=47%
HALLMARK_ANDROGEN_RESPONSE	90	0.17	1.9	0.011	0.021	tags=77%, list=60%, signal=188%
HALLMARK_BILE_ACID_METABOLISM	90	0.17	1.89	0.018	0.021	tags=37%, list=20%, signal=46%
HALLMARK_APICAL_JUNCTION	166	0.11	1.72	0.02	0.044	tags=45%, list=34%, signal=67%
HALLMARK_HYPOXIA	175	0.11	1.73	0.027	0.044	tags=46%, list=35%, signal=69%

Table S4. Hallmark gene sets negatively enriched in MV4-11-CR cells

HALLMARK	Size	ES	NES	NOM p-value	FDR q-value	Leading edge
HALLMARK_EPITHELIAL_MESENCHYMAL_TRANSITION	158	-0.27	-3.93	0	0	tags=56%, list=29%, signal=78%
HALLMARK_KRAS_SIGNALING_DN	125	-0.27	-3.5	0	0	tags=54%, list=28%, signal=75%
HALLMARK_INFLAMMATORY_RESPONSE	168	-0.19	-2.91	0	0	tags=55%, list=36%, signal=86%
HALLMARK_TNFA_SIGNALING_VIA_NFKB	185	-0.18	-2.87	0	0	tags=63%, list=45%, signal=114%
HALLMARK_INTERFERON_GAMMA_RESPONSE	189	-0.16	-2.48	0	0.001	tags=62%, list=47%, signal=114%
HALLMARK_MYOGENESIS	162	-0.16	-2.47	0	0.001	tags=55%, list=39%, signal=89%
HALLMARK_APICAL_JUNCTION	167	-0.15	-2.33	0	0.003	tags=43%, list=27%, signal=58%
HALLMARK_COAGULATION	104	-0.18	-2.19	0.002	0.007	tags=45%, list=27%, signal=62%
HALLMARK_ALLOGRAFT_REJECTION	167	-0.15	-2.18	0	0.007	tags=46%, list=31%, signal=65%
HALLMARK_KRAS_SIGNALING_UP	158	-0.12	-1.83	0.01	0.046	tags=42%, list=29%, signal=59%
HALLMARK_COMPLEMENT	177	-0.12	-1.76	0.023	0.057	tags=49%, list=37%, signal=76%
HALLMARK_ESTROGEN_RESPONSE_EARLY	173	-0.11	-1.75	0.019	0.054	tags=45%, list=34%, signal=67%
HALLMARK_ESTROGEN_RESPONSE_LATE	165	-0.11	-1.71	0.017	0.059	tags=44%, list=32%, signal=64%
HALLMARK_BILE_ACID_METABOLISM	94	-0.15	-1.7	0.031	0.058	tags=53%, list=39%, signal=86%

HALLMARK_APOPTOSIS	147	-0.12	-1.67	0.036	0.061	tags=52%, list=40%, signal=86%
HALLMARK_IL6_JAK_STAT3_SIGNALING	74	-0.17	-1.66	0.038	0.059	tags=53%, list=36%, signal=82%
HALLMARK_IL2_STAT5_SIGNALING	176	-0.11	-1.64	0.033	0.063	tags=63%, list=52%, signal=131%
HALLMARK_UV_RESPONSE_DN	133	-0.12	-1.64	0.04	0.06	tags=62%, list=51%, signal=125%
HALLMARK_ANGIOGENESIS	27	-0.25	-1.61	0.037	0.064	tags=52%, list=27%, signal=71%
HALLMARK_HYPOXIA	180	-0.1	-1.58	0.037	0.069	tags=34%, list=24%, signal=45%

Table S5. Hallmark gene sets negatively enriched in MOLM-13-CR cells

**HALLMARK**	**Size**	**ES**	**NES**	**NOM p-value**	**FDR q-value**	**Leading edge**
HALLMARK_MYC_TARGETS_V1	200	-0.3	-4.88	0	0	tags=87%, list=57%, signal=198%
HALLMARK_E2F_TARGETS	199	-0.2	-3.27	0	0	tags=91%, list=72%, signal=321%
HALLMARK_MYC_TARGETS_V2	58	-0.36	-3.2	0	0	tags=84%, list=49%, signal=163%
HALLMARK_OXIDATIVE_PHOSPHORYLATION	198	-0.19	-3.14	0	0	tags=83%, list=64%, signal=227%
HALLMARK_EPITHELIAL_MESENCHYMAL_TRANSITION	147	-0.22	-3.14	0	0	tags=35%, list=13%, signal=39%
HALLMARK_DNA_REPAIR	148	-0.19	-2.72	0	0	tags=85%, list=66%, signal=251%
HALLMARK_UNFOLDED_PROTEIN_RESPONSE	112	-0.18	-2.28	0	0.002	tags=79%, list=61%, signal=198%
HALLMARK_ADIPOGENESIS	183	-0.11	-1.75	0.018	0.035	tags=79%, list=68%, signal=246%
HALLMARK_MTORC1_SIGNALING	196	-0.1	-1.63	0.047	0.057	tags=87%, list=77%, signal=379%

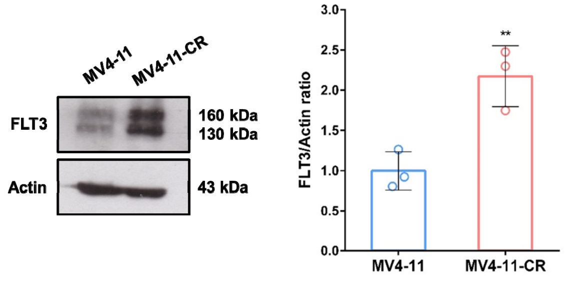

(a)

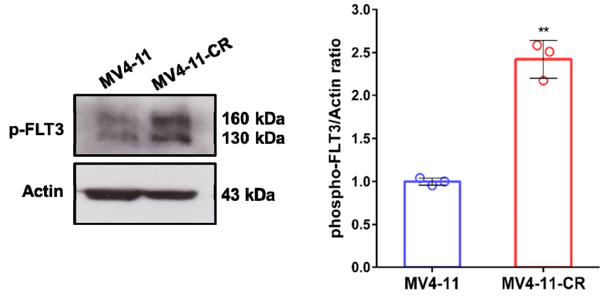

(b)

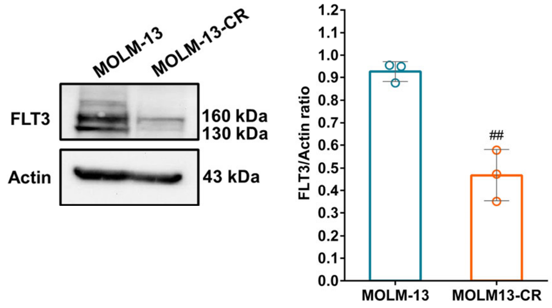

(c)

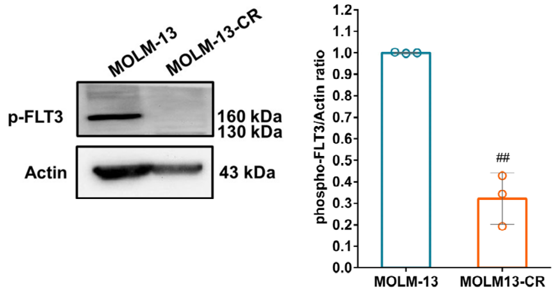

(d)

Figure S1. FLT3 expression and phosphorylation in cytarabine-resistant AML cells. Western blot analysis and quantification of total FLT3 and phosphorylated FLT3 in **(a, b)** MV4-11 and MV4-11-CR cells and **(c, d)** MOLM-13 and MOLM-13-CR cells. Representative blots are shown. Data are presented as the mean ± SD of three independent experiments, with $^{**}p < 0.01$ indicating significant differences compared to MV4-11 and $^{\#\#}p < 0.01$ indicating significant differences compared to MOLM-13 cells.

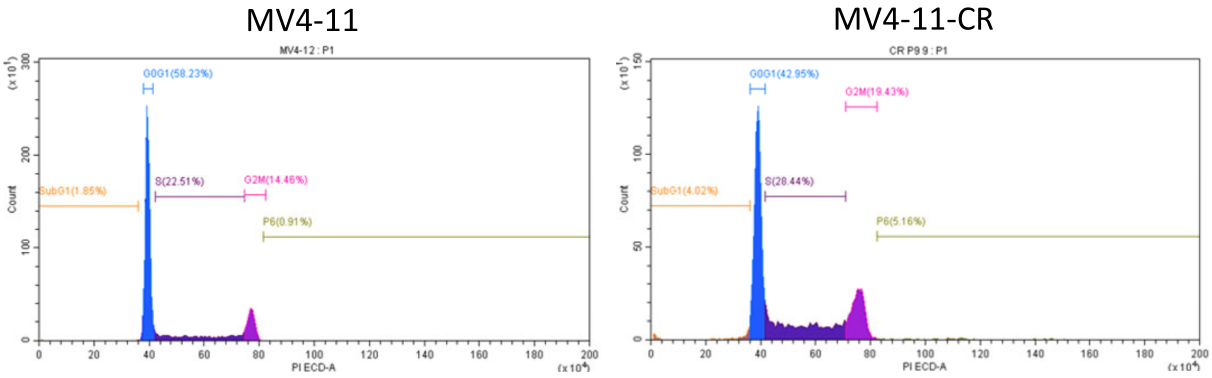
MV4-11
MV4-11-CR
G0G1(58.23%)
S(22.51%)
G2M(14.46%)
SubG1(1.85%)
P6(0.91%)
G0G1(42.95%)
G2M(19.43%)
S(28.44%)
SubG1(4.02%)
P6(5.16%)
Count
PI ECD-A

(a)

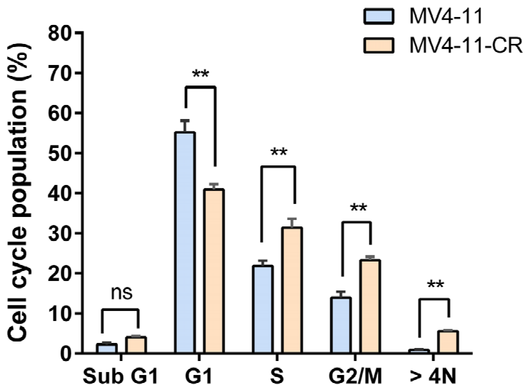
MV4-11
MV4-11-CR
Cell cycle population (%)
ns
**
Sub G1
G1
S
G2/M
> 4N

(b)

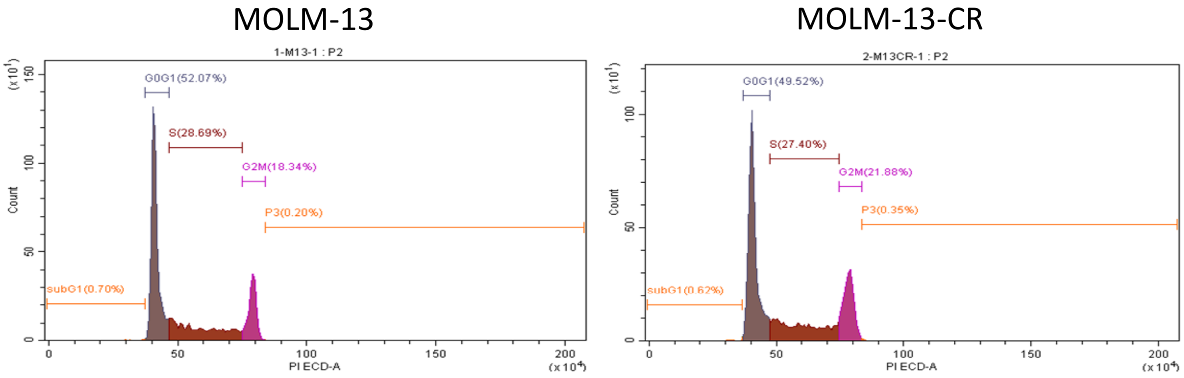
MOLM-13
MOLM-13-CR
G0G1(52.07%)
S(28.69%)
G2M(18.34%)
P3(0.20%)
subG1(0.70%)
G0G1(49.52%)
S(27.40%)
G2M(21.88%)
P3(0.35%)
subG1(0.62%)
Count
PI ECD-A

(c)

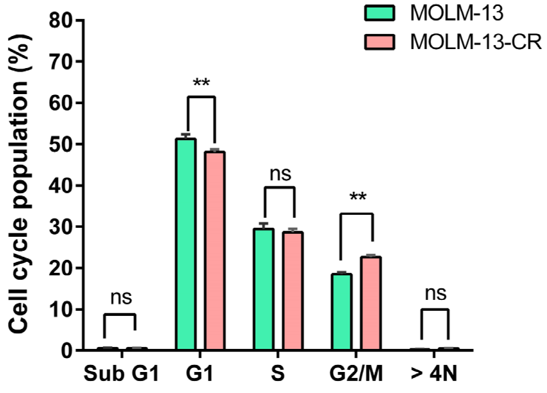
MOLM-13
MOLM-13-CR
Cell cycle population (%)
ns
**
Sub G1
G1
S
G2/M
> 4N

(d)

Figure S2. Cell-cycle distribution in MV4-11, MV4-11-CR, MOLM-13, and MOLM-13-CR cells. Cell-cycle and DNA-content distributions were analyzed by propidium iodide staining and flow cytometry. **(a)** Representative histograms of cell cycle distribution in MV4-11 and MV4-11-CR cells. **(b)** Quantification of Sub-G1, G1, S, G2/M and >4N populations in MV4-11 and MV4-11-CR cells. **(c)** Representative histograms of cell cycle distribution in MOLM-13 and MOLM-13-CR cells. **(d)** Quantification of Sub-G1, G1, S, G2/M and >4N populations in MOLM-13 and MOLM-13-CR cells. Data are presented as the mean ± SD of three independent experiments, with $^{**}p < 0.01$ indicating significant differences compared with the respective parental cells; ns, not significant.

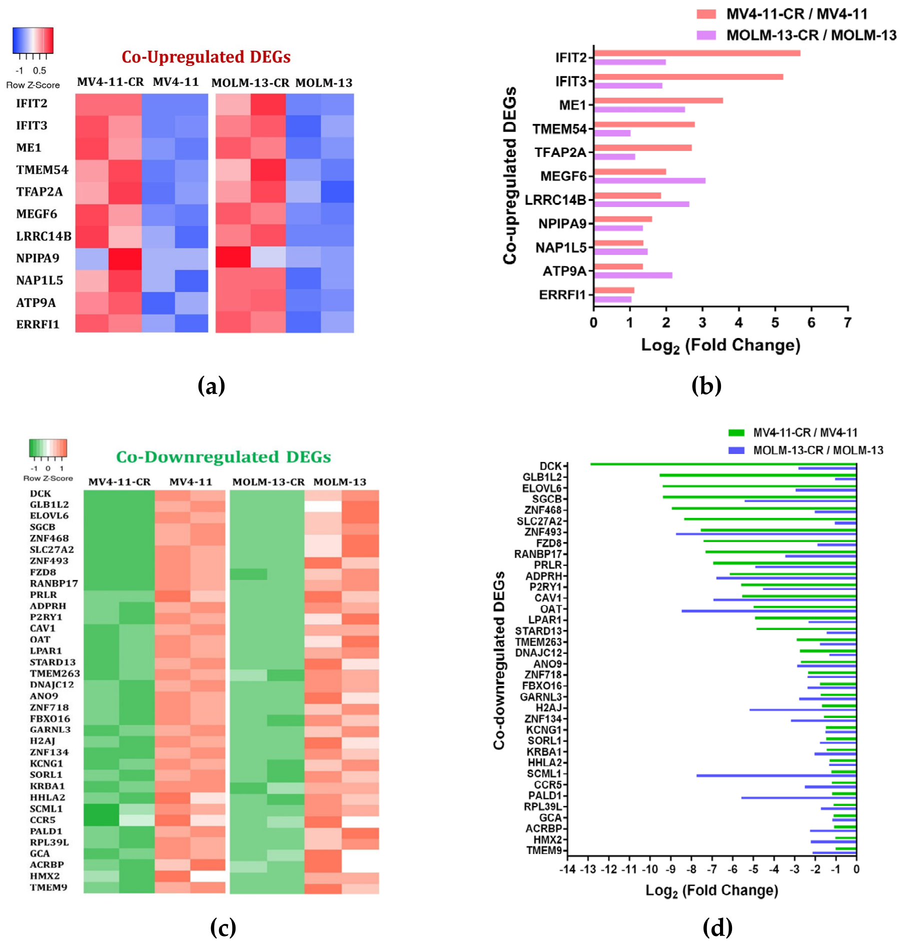

Figure S3. The co-upregulated and co-down-regulated DEGs in MV4-11-CR and MOLM-13-CR cells. (a) Heatmap of co-upregulated DEGs. (b) log2 fold change values of co-upregulated DEGs. (c) Heatmap of co-downregulated DEGs. (d) log2 fold change values of co-downregulated DEGs.

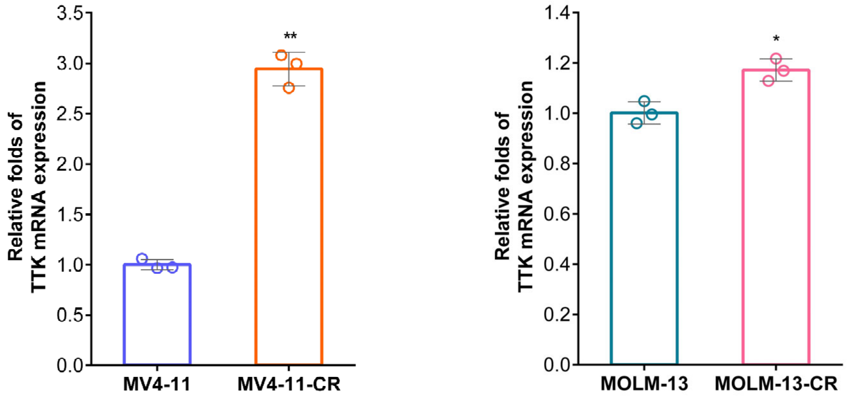

(a) **(b)**

Figure S4. TTK mRNA expression in MV4-11, MV4-11-CR, MOLM-13, and MOLM-13-CR cells. *TTK* mRNA levels were measured using RT-qPCR in **(a)** MV4-11 and MV4-11-CR cells; **(b)** MOLM-13 and MOLM-13-CR cells. RT-qPCR data are presented as mean ± SD from at least three independent experiments. *$p < 0.05$ and **$p < 0.01$ indicate significant differences compared to the respective parental MV4-11 or MOLM-13 control groups.
